# Metagenomic next-generation sequencing for cryptococcal meningitis diagnosis: a single-center experience

**DOI:** 10.3389/fcimb.2025.1626290

**Published:** 2025-12-11

**Authors:** Zichun Zhao, Yu Zhang, Jinsheng Fu, Lili Yu

**Affiliations:** Department of Neurology, The Second Hospital of Hebei Medical University, Shijiazhuang, Hebei, China

**Keywords:** cryptococcal meningitis, metagenomic next-generation sequencing, fungal infection, rapid diagnostics, immunocompromised

## Abstract

**Purpose:**

Cryptococcal meningitis (CM) is a devastating central nervous system infection with substantial mortality, particularly when diagnosis is delayed. This study aims to evaluate the diagnostic performance of metagenomic next-generation sequencing (mNGS) for CM detection in comparison with conventional tests.

**Methods:**

We enrolled 23 consecutive patients with suspected CM at a tertiary center. All patients met a composite reference standard (CRS) based on CSF cryptococcal antigen (CrAg), CSF/sterile-site culture for Cryptococcus, or CNS histopathology; mNGS was excluded from the CRS. Primary outcomes were CRS-based sensitivity (computed only among CRS-positive patients who underwent each assay) and turnaround times (TATs); pairwise agreement metrics (PPA/NPA) between mNGS and conventional assays were estimated in co-tested subsets.

**Results:**

mNGS identified Cryptococcus in 18/23 (78.3%) cases and detected viral co-pathogens (EBV/CMV/HIV-1) in 5 patients. CRS-based sensitivities were: CrAg LFA (CSF) 83.3% (5/6), Alcian blue 72.7% (16/22), India ink 50.0% (3/6), and CSF culture 66.7% (8/12). Pairwise agreement favored mNGS against culture and CrAg (e.g., PPA 100% vs culture 8/8] and vs CSF CrAg [5/5]), with limited NPA where denominators were small. Median (IQR) TATs were 0.5 (0.5–0.5) days for CrAg LFA, 1 (0.5–1) day for India ink, 5 (3–8) days for first positive culture, and 2 (1–4) days for mNGS.

**Conclusion:**

CSF mNGS complements CrAg, microscopy, and culture by increasing Cryptococcus detection and revealing mixed infections, with particular utility in atypical, pretreated, or complex hosts. Larger studies are warranted to validate clinical utility and define optimal integration with existing workflows.

## Introduction

Cryptococcal meningitis (CM) is the most common fungal infection of the central nervous system (CNS) and disproportionately affects immunocompromised individuals, especially those infected with human immunodeficiency virus (HIV) (Temfack et al., 2021; McHale et al., 2023). Globally, CM causes an estimated 223,100 cases annually, with 73% of cases occurring in sub-Saharan Africa and 19% in Asia and the Pacific regions (Rajasingham et al., 2022). In China, however, most patients are HIV-negative, and 55% to 67% of cases occur in immunocompetent individuals (Zhang et al., 2020), a pattern that complicates timely recognition and contributes to worse outcome. If untreated, CM is almost uniformly fatal, even with antifungal therapy, mortality remains high (rate, 10% to 40%) and recurrence is frequent (rate, 20% to 25%) (Wang et al., 2021). Delayed diagnosis is the most common - and preventable - driver of poor prognosis, highlighting the need for accurately, timely and widely applicable diagnostics.

Cerebrospinal fluid (CSF) culture remains the conventional gold standard, yet suffers from prolonged turnround time (Cano et al., 2020; Chang et al., 2024). India ink staining offers a fast and low-cost alternative, but has variable sensitivity (initial positivity 42%-86%) and often requires repeated sampling (Kwizera et al., 2024). Immunodiagnostics have reshaped care: cryptococcal antigen (CrAg) assays—especially lateral-flow assays (LFAs)—are recommended first-line tests in suspected CM and for screening in advanced HIV disease; pooled estimates show excellent accuracy (≈ 99%/99% in CSF and ≈ 96%/96% in serum) (Ford et al., 2018; WHO Guidelines Approved by the Guidelines Review Committee, 2022; Macrae et al., 2023; Chang et al., 2024).

However, false-negative CrAg results can occur in specific settings (e.g., post-zone/prozone “hook” effects at very high antigen levels, very low fungal burden, or capsule-deficient strains), and prior antifungal exposure frequently reduces culture yield (Kojima et al., 2018; Shende et al., 2022; Skipper et al., 2022; Zimmet et al., 2023).

Against this backdrop, metagenomic next-generation sequencing (mNGS) offers hypothesis-free, high-throughput pathogen detection directly from CSF (Wilson and Tyler, 2022; Su et al., 2024). We considered mNGS complementary—rather than a replacement—to CrAg and conventional methods. *A priori*, we expected mNGS to add value by (i) detecting mixed infections and alternative etiologies in immunocompromised or otherwise atypical hosts, (ii) providing species/lineage-level resolution (e.g., distinguishing C. neoformans from C. gattii and identifying unusual taxa), and (iii) remaining informative when conventional assays are limited by pretreatment or immunoassay interference (Gan et al., 2022; Benoit et al., 2024).

We therefore conducted a prospective case series at a tertiary center in China to assess the diagnostic yield of CSF mNGS in suspected CM in parallel with conventional methods.

## Materials and methods

### Study design and setting

This prospective case series included 23 consecutive patients with clinical suspicion of CM admitted to the Department of Neurology, Second Hospital of Hebei Medical University, a tertiary referral center in Hebei Province, China, between October 10, 2020 and December 31, 2023. The study was conducted as part of a broader research initiative applying mNGS to CSF for etiologic diagnosis of CNS infections.

Informed written consent was obtained from all participants. The study protocol was approved by the Institutional Ethics Committee (Approval No. 2020-R527) and complied with the Declaration of Helsinki.

### Diagnostic criteria for CM suspects

Patients were considered have suspected CM if they presented with any combination of: 1, subacute or chronic headache, often lasting for several days to weeks, and not responsive to standard analgesics; 2, neurological symptoms or signs such as fever, altered mental status (e.g., confusion, lethargy, or personality changes), cranial nerve palsies, seizures, or increased opening pressure (e.g., vomiting, papilledema); 3, CSF abnormalities, including lymphocytic pleocytosis, elevated opening pressure (consistent with raised intracranial pressure), low glucose levels, and elevated protein concentration; and/or 4, immunocompromised status (e.g., HIV infection, organ transplantation, corticosteroid use, or chemotherapy). In some instances, no predisposing condition was identified.

### Reference standard

In the absence of a single perfect gold standard for CM, we used a composite reference standard (CRS): CM present if any CSF-based or sterile-site conventional evidence was positive—CSF CrAg, CSF/sterile-site culture for Cryptococcus, or CNS histopathology—with mNGS excluded to avoid incorporation bias (Bossuyt et al., 2015; Chang et al., 2024).

### Diagnostic pathway at our institution

At presentation, patients with suspected CM underwent neuroimaging as clinically indicated and diagnostic lumbar puncture. Initial CSF studies included opening pressure, cell count and differential, protein and glucose, cytology/smear, staining (India ink or Alcian blue), fungal culture. Cryptococcal capsular antigen (CrAg) testing was available and performed at the treating clinician’s discretion. In parallel with conventional tests, an additional CSF aliquot (≈2 mL) was reserved for metagenomic next-generation sequencing (mNGS) and stored at −80 °C within 30 minutes of collection, and shipped on dry ice to the reference laboratory. mNGS was typically ordered concurrently when CM was suspected, particularly when atypical hosts or mixed infections were considered, or when early organism-level identification could influence management. Laboratory reports for stains and routine chemistries were available same day; culture results were issued as they became available. The external laboratory returned mNGS results as written electronic reports to the ordering clinicians, who integrated results into care at their discretion.

CrAg testing was available throughout the study period via a commercial lateral-flow immunochromatographic assay (LFA) implemented in the hospital laboratory. Specimen types included CSF and/or serum, selected according to clinical context. Positivity was defined per the manufacturer’s instructions for use (appearance of a reactive test line within the specified read time). When clinical suspicion remained high despite an initial non-reactive result, dilutional testing could be performed at the clinician’s discretion to mitigate potential high-antigen (post-zone/prozone) effects. CrAg was not mandated for all patients; in our cohort it was performed for a subset.

Turnaround time (TAT) was defined as the interval from CSF collection to the first actionable laboratory report for each modality: typically, same-day direct stains (India ink/Alcian blue), CrAg LFA, the first positive fungal culture report (or final negative report when no growth), and the written mNGS report issued by the reference laboratory.

### DNA extraction and library preparation

All CSF samples were shipped on dry ice to Hugobiotech (Beijing, China) and processed on the PACEseq platform. Genomic DNA was extracted using the QIAamp DNA Micro Kit (Qiagen, Germany) according to the manufacturer’s protocol. Libraries were constructed with the QIAseq Ultralow Input Library Kit and assessed for quality by Agilent 2100 Bioanalyzer and Qubit 2.0 fluorometer. Sequencing was performed on the Illumina NextSeq 550 (Illumina, USA).

### Bioinformatics and interpretation

Raw sequencing data were processed with an in-house pipeline. Adapters were trimmed and reads failing quality or complexity filters were removed; PCR/optical duplicates were collapsed, and short reads (< 35 bp) were excluded. Remaining reads were aligned to the human reference genome (hg38) to subtract host-derived sequences. Non-human reads were then aligned against a curated snapshot of the NCBI microbial genome databases (bacteria, fungi, viruses, parasites) using the Burrows–Wheeler Aligner (BWA). Ambiguous alignments were discarded (mapping quality ≥ 30, alignment length ≥ 50 bp, identity ≥ 95%). Taxa were quantified as unique species-level reads and reads per million (RPM).

To control for batch-specific background and index hopping, species calls were made using pre-specified rules tied to the no-template control (NTC) processed in each batch. For viruses, Mycobacterium tuberculosis, and Cryptococcus, a sample was called positive if it contained ≥ 1 unique species-level read and exceeded background as follows: when the NTC had 0 reads for that species, the sample was called positive; when the NTC had ≥ 1 read, positivity additionally required RPM_sample/RPM_NTC > 5. For other bacteria, fungi, and parasites, positivity required either top-10 rank within its microbial class and absence in the NTC, or RPM_sample/RPM_NTC > 10 when NTC RPM ≠ 0. These thresholds were defined *a priori* based on internal validation and published practice to minimize false positives from low-level background.

Library preparation used unique dual indexes (UDI) with non-reused index pairs; pre-PCR and post-PCR steps were performed in physically separated rooms with dedicated hoods and aerosol-resistant tips. Each batch included an extraction blank and a no-template library control (NTC) processed alongside specimens. Computationally, we removed low-complexity reads, deduplicated alignments, and flagged taxa present in the NTC and lacking multi-locus support. Putative cross-sample carryover was reviewed at the specimen→run→batch levels before any positive call.

### Criteria for a positive mNGS result

For bacteria (excluding *Mycobacterium tuberculosis*), fungi (excluding *Cryptococcus*), and parasites: detection was considered positive if the organism ranked among the top 10 microbes of its class and was absent in the negative control (NTC), or if RPM_sample/RPM_NTC > 10 when NTC RPM ≠ 0. For viruses, *Mycobacterium tuberculosis* and *Cryptococcus*, positivity required at least one unique species-level read, absent in NTC or RPM_sample/RPM_NTC > 5 when NTC RPM ≠ 0.

## Results

### General characteristics

**Figure 1:**
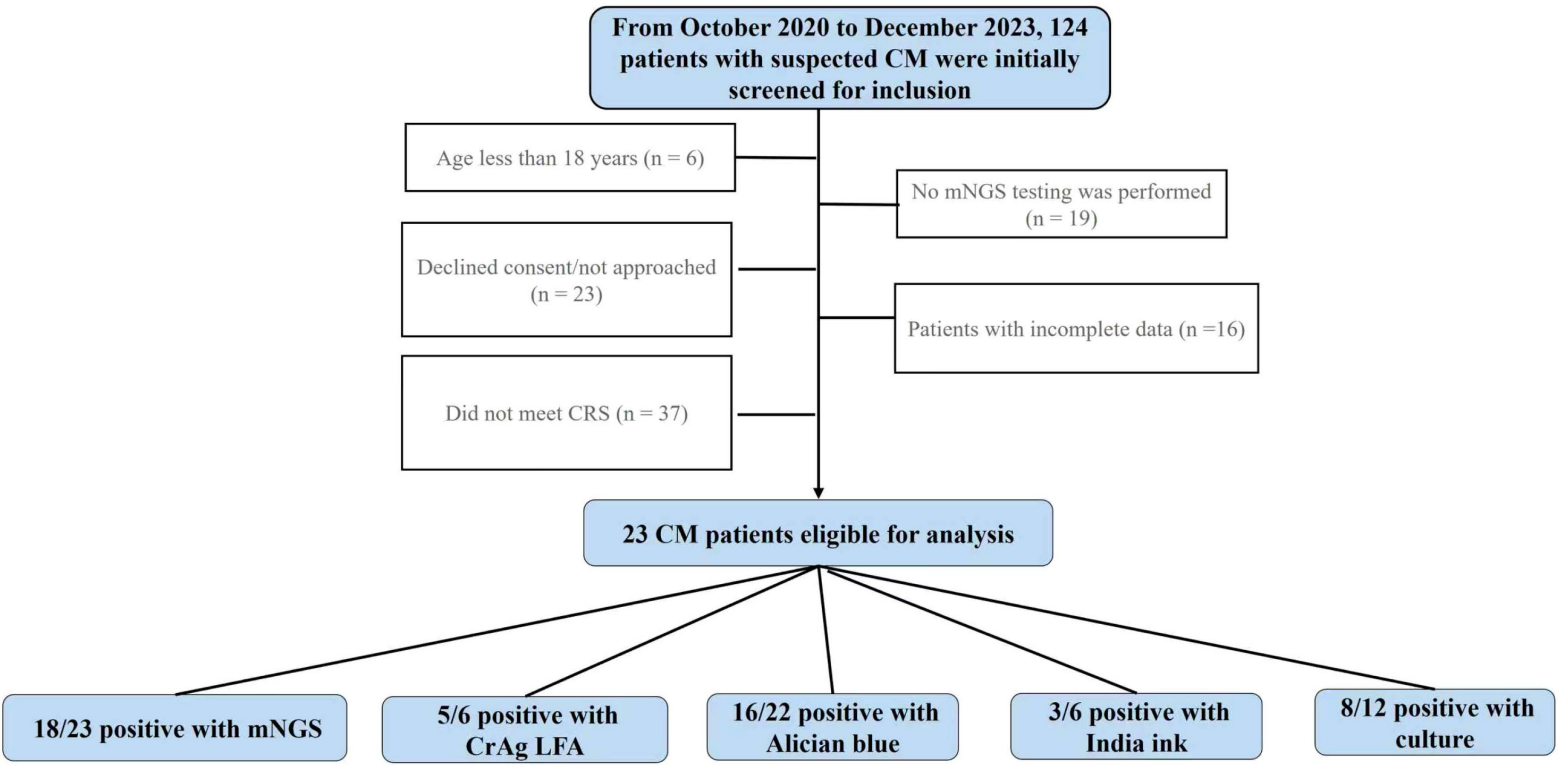
Flowchart of study participants.

A flow diagram summarizes screening, inclusion/exclusion, index (mNGS) and comparator test uptake, and reasons for non-performance (**Figure 1**). Among the included 23 patients (16 men and 7 women; median age, 55 years; range, 33–71 years), 18 (78.3%) had underlying conditions, including Nephrotic syndrome (6 cases, 26.1%), type 2 diabetes mellitus (DM) (3 cases, 13.0%), chronic hepatitis B (2 cases, 8.7%), systemic lupus erythematosus (SLE), rheumatoid arthritis (RA), HIV infection each with 1 case (4.3%); other immunosuppressive or chronic conditions, such as interstitial lung disease, bone marrow disorders, syphilis, Evans syndrome, and post-splenectomy status, were also noted (4 cases, 17.4%) (**Table 1**).

**Table 1:** Clinical characteristics at presentation and imaging findings in 23 patients with cryptococcal meningitis.

ID	Sex	Age	Headache	Fever	Meningeal signs	Focal neurological deficits	Altered consciousness	Seizures	Underlying conditions	Chest CT findings	Brain MRI findings
1	Male	58	Yes	Yes	No	No	No	No	Pneumoconiosis	Not examined	Parenchymal lesion
2	Female	45	Yes	No	No	No	No	No	SLE, nephritis	Nodules	None
3	Female	66	Yes	No	Yes	No	No	No	Multiple myeloma	Nodules	Acute cerebral infarction
4	Male	38	Yes	No	No	No	No	No	Ulcerative colitis	Nodules, Possible special infection	Parenchymal lesion, Meningitis
5	Female	55	Yes	No	Yes	Yes	Yes	Yes	nephrotic syndrome, Rheumatoid arthritis	Nodules	Acute cerebral infarction
6	Female	57	Yes	Yes	No	No	Yes	No	Nephrotic syndrome, Type 2 diabetes mellitus	Mild inflammation	Acute cerebral infarction
7	Male	53	No	Yes	Yes	Yes	No	No	Hepatitis B, Type 2 diabetes mellitus, Splenectomy	Chronic inflammation	Acute cerebral infarction
8	Female	60	No	Yes	No	No	No	No	None	Mild inflammation	Meningitis
9	Male	47	Yes	Yes	Yes	No	No	No	None	None	Parenchymal lesion, Meningitis
10	Male	71	No	No	No	No	No	No	Nephropathy, Liver disease	None	None
11	Male	33	Yes	Yes	No	No	No	No	None	None	None
12	Male	57	Yes	No	Yes	No	No	No	Type 2 diabetes mellitus	None	Parenchymal lesion, Meningitis
13	Male	51	Yes	Yes	No	Yes	No	No	Evans syndrome	Multiple small nodules	None
14	Female	71	No	Yes	No	Yes	Delayed response	No	Nephropathy syndrome	Small airway inflammation	Meningitis
15	Male	47	Yes	Yes	Yes	No	No	No	Nephropathy syndrome	Mild inflammation	Parenchymal lesion
16	Male	47	Yes	Yes	No	Yes	No	No	Immunosuppressive therapy	Suspicious for fungal infection	None
17	Male	46	Yes	Yes	Yes	No	No	No	Interstitial pneumonia	Not examined	Meningeal involvement
18	Male	37	Yes	Yes	Yes	Yes	No	No	syphilis, HIV	Nodules with cavitary changes	Acute cerebral infarction
19	Female	54	Yes	Yes	Yes	No	No	No	Type 2 diabetes mellitus	Increased pulmonary markings	Meningeal involvement
20	Male	56	No	No	No	Yes	No	No	None	Multiple metastatic lesions in both lungs	Thoracic spinal cord lesion
21	Male	56	Yes	Yes	Yes	No	No	Yes	Nephropathy syndrome, Oral corticosteroids	Inflammation in right and lower left lung	None
22	Male	55	Yes	Yes	No	Yes	No	No	Hepatitis B, Nephropathy syndrome, Oral corticosteroids	Right lower lobe pulmonary nodules	None
23	Male	16	Yes	Yes	Yes	Yes	No	No	None	No abnormalities	Parenchymal lesion

Per-patient demographics, symptoms/signs at admission, underlying conditions, and major radiologic findings. “Yes/No” indicates presence or absence at presentation. Chest CT categories summarize dominant patterns (e.g., nodules, inflammatory changes, suspected fungal lesions). Brain MRI categories reflect the main neuroradiologic impression (parenchymal lesion, meningeal enhancement/involvement, acute cerebral infarction, spinal cord lesion), or “None” when no inflammatory abnormalities were identified. “Not examined” indicates the modality was not performed.

CT, computed tomography; MRI, magnetic resonance imaging; HIV, human immunodeficiency virus; SLE, systemic lupus erythematosus; DM, diabetes mellitus.

Presentations were acute (n = 4), subacute (n = 16), or chronic (n = 3). Three patients (Cases 5-7) were severely affected (modified Rankin Scale (mRS) score of 5). Headache (73.9%) and fever (69.6%) were the most common symptoms; meningeal signs were present in 47.8% of patients, and focal neurological deficits in 39.1%, including limb weakness and dysarthria (**Table 1**).

### Imaging findings

Chest CT was performed in 21 patients, among whom 17 (81.0%) had abnormal pulmonary findings, including pulmonary nodules, localized inflammation, or suspected infectious lesions. Pulmonary lesions in two cases were suspected to be associated with acute cryptococcal infection, while one lesion, initially suspected to be metastatic, was confirmed as cryptococcal by CT-guided biopsy (**Table 1**).

All patients underwent brain MRI. Cranial MRI revealed parenchymal lesions in 3 patients, combined parenchymal and meningeal involvement in 3, inflammation-related acute infarcts in 5, isolated meningeal enhancement in 4, thoracic spinal cord involvement in 1, and no inflammatory abnormalities in 7 (**Table 1**).

### Laboratory findings

Thirteen patients (56.5%) had elevated opening pressure (>200 mmH_2_O). CSF leukocytosis was present in 21 cases, with WBC counts of 5 to 540/mm³; 9 had <100/mm³ and 12 had ≥100/mm³. Protein was elevated in 22 cases, and glucose was decreased in 9 cases (0.1–2.09 mmol/L).

Microbiological confirmation of cryptococcal infection was achieved via Alcian blue staining (n=16), smear (n=6), culture (n=8), antigen testing (n=5), or lung biopsy in 1 case with thoracic spinal cord involvement) (**Table 2**).

**Table 2:** Cerebrospinal fluid laboratory findings and mNGS results for 23 patients with cryptococcal meningitis.

ID	CSF opening pressure (mmH2O)	CSF WBC (/mm³)	CSF protein (g/L)	CSF glucose (mmol/L)	Cytology findings	Culture/Antigen	Time to mNGS (days)	Cryptococcus reads	Coverage	Relative abundance (%)	Depth	Human background (%)
1	150	207	3.9	1.18	Scattered cryptococci and phagocytes	Positive capsular antigen in CSF and blood	64 days	8	0.00	0.04	1.16	96.98
2	>330	30	0.9	3.14	Clustered cryptococci	Culture indicates *Cryptococcus albidus*	23 days	2	0.00	0.00	1.44	65.40
3	200	15	0.56	0.1	Clustered cryptococci	Suspected cryptococci on CSF routine test	30 days	2714	0.97	1.84	1.17	96.48
4	275	100	0.96	1.71	Not examined	Positive cryptococcal antigen in CSF	35 days	3	0.00	0.00	1	74.90
5	180	16	0.42	2.87	Clustered cryptococci	Not examined	20 days	5344	1.25	11.81	1.19	84.01
6	>330	220	1.32	2.09	Predominantly neutrophilic response	CSF culture positive for Cryptococcus neoformans	21 days	150	0.06	0.44	1.14	91.21
7	250	540	1.28	5.28	Cryptococci observed	CSF culture positive for Cryptococcus neoformans	20 days	1971	0.71	1.70	1.16	99.44
8	230	450	0.81	3.51	None	Cryptococcus observed on smear	35 days	1	0.00	0.00	1	96.88
9	250	26	0.6	3.16	Several cryptococci observed	CSF culture positive for *Cryptococcus neoformans*	90 days	4	0.00	0.00	1	66.90
10	180	12	0.52	4.84	Single cryptococcus observed	Not examined	1 year	Negative				
11	230	270	0.6	2.83	Several *Cryptococcus* organisms observed	Not examined	10 days	Negative				
12	140	5	0.64	4.86	Lymphocyte predominance with 2% neutrophils, cryptococci observed	Positive India ink staining	1 year, 1 month, and 20 days	Negative				
13	>330	100	1.9	2.83	Clustered cryptococci	CSF culture positive for Cryptococcus neoformans	30 days	2484	0.85	0.20	1.37	66.56
14	50	81	1.01	1.44	Predominantly lymphocytes	Positive serum cryptococcal antigen	15 days	6	0.01	0.00	1	95.62
15	150	101	0.45	3.5	Lymphocytic response	Positive India ink staining	60 days	Negative				
16	195	18	0.72	4.4	Several cryptococci observed	Blood culture was positive for *Cryptococcus neoformans*; cerebrospinal fluid (CSF) culture yielded *Staphylococcus epidermidis*; cryptococcal antigen was detected in CSF, and EBV PCR positive	47 days	350	0.08	0.36	2.06	81.25
17	>330	117	2.0	0.41	Multiple clusters of cryptococci	Not examined	21 days	14	0.00	0.01	1.37	82.63
18	>330	150	0.6	1.29	Cryptococcal spores	Cryptococcal capsule visualized on CSF smear	60 days	108	0.03	0.10	1.41	99.34
19	310	0	0.4	3.3	Clustered cryptococci	CSF culture positive for *Cryptococcus neoformans*	100 days	28	0.01	0.10	1.00	70.23
20	30	0	0.3	3.3	No abnormalities	Cryptococcal infection suspected by lung biopsy	60 days	Negative				
21	240	190	1.8	2.59	Scattered *Cryptococcus* organisms	Not examined	30 days	200	0.05	0.20	1.61	93.00
22	>330	56	1.87	0.16	Lymphocytic response	Positive India ink staining	16	168	0.06	0.08	1	83.75
23	120	161	3.13	1.42	Abnormal CSF cytology with a single cryptococcal cell	Positive capsular antigen	60	4	0.75	1.84	1.19	85.07

CSF opening pressure, leukocyte count, protein, and glucose at diagnostic lumbar puncture, together with cytology and conventional microbiology results, the interval from symptom onset to mNGS sampling (“Time to mNGS”), and mNGS metrics (species-specific reads, coverage, relative abundance, sequencing depth, and proportion of human background reads). “Negative” indicates no organism meeting predefined positivity criteria (see Methods). Time to mNGS (days), from symptom onset to sampling.

Cytology Findings, from Alician blue and cytological classification.

Opening pressure reported in mmH_2_O; WBC as cells/mm³; protein in g/L; glucose in mmol/L. Coverage and relative abundance are expressed as percentages; depth reflects average sequencing depth for the target; human background is the percentage of host reads in the library.

CSF, cerebrospinal fluid; mNGS, metagenomic next-generation sequencing; EBV, Epstein–Barr virus.

Turnaround times (TATs) were a median of 0.5 days (interquartile range, IQR, 0.5 to 0.5) for CrAg LFA; 1 day (0.5 to 1) for India ink; and a median of 5 days (interquartile range, IQR 3–8) to the first positive CSF culture report, respectively.

### mNGS results

The mean interval from symptom onset to mNGS was 71 days (range: 10–415 days) and the turnaround time was a median of 2 days (IQR, 1 to 4). The Cryptococcus was successfully identified by mNGS in 18/23 cases (78.26%).

Among positives, the average number of species-specific reads was 590 (range: 1–5344), with a mean relative abundance of 1.09% (range: 0.00–11.81%). Human background reads averaged 85.05% (range: 65.40–99.44%) (**Table 2**).

Viral sequences were identified in 5 patients (EBV/HHV-4 in four; cytomegalovirus (CMV) in one; HIV-1 and torque teno virus in one each) (**Table 3**).

**Table 3:** Concurrent pathogen detection by mNGS in patients with cryptococcal meningitis.

Patient ID	Fungal pathogen (Reads)	Viral pathogen(s) (Reads)
2	Cryptococcus neoformans (8)	Cytomegalovirus (4);Epstein–Barr virus (24)
5	Cryptococcus neoformans (5344)	Epstein–Barr virus (4)
9	Cryptococcus neoformans (4)	Epstein–Barr virus (10)
16	Cryptococcus neoformans (350)	Epstein–Barr virus (2), also positive EBV PCR in blood
18	Cryptococcus neoformans (108)	HIV-1 (4);Torque teno virus (32)

Co-detected organisms and corresponding unique reads in CSF by mNGS among patients with confirmed cryptococcal meningitis. Viral detections include EBV, cytomegalovirus, HIV-1, and torque teno virus. Read counts denote species-specific mapped reads after human subtraction and quality filtering.

EBV, Epstein–Barr virus; HIV-1, human immunodeficiency virus type 1; mNGS, metagenomic next-generation sequencing.

### Diagnostic performance across assays (CRS sensitivity and PPA/NPA)

The observed sensitivities vs CRS were calculated only among CRS-positive patients who underwent the index and 95% confidence intervals were computed using the Wilson score method. The results were: CrAg LFA (CSF) 83.3% (5/6; 95% CI 43.6–97.0), Alcian blue 72.7% (16/22; 95% CI 51.8–86.8), India ink 50.0% (3/6; 95% CI 18.8–81.2), and CSF culture 66.7% (8/12; 95% CI 39.1–86.2).

Pairwise agreement between mNGS and conventional tests in the co-tested subset was as follows: CrAg LFA (CSF)—PPA 100.0% (5/5; 95% CI, 56.6–100) and NPA 100.0% (1/1; 95% CI, 20.7–100); India ink—PPA 33.3% (1/3; 95% CI, 6.1–79.2) and NPA 66.7% (2/3; 95% CI, 20.8–93.9); Alcian blue (CSF smear)—PPA 94.1% (16/17; 95% CI, 73.0–99.0) and NPA 60.0% (3/5; 95% CI, 23.1–88.2); and CSF culture—PPA 100.0% (8/8; 95% CI, 67.6–100) and NPA 25.0% (1/4; 95% CI, 4.6–69.9).

## Discussion

In this prospective consecutive series, CSF mNGS increased the diagnostic yield for cryptococcal meningitis (CM) and uncovered viral co-pathogens in a subset, supporting its role as a complement—rather than a replacement—to conventional testing. Notably, we report CRS-based sensitivities for each assay and pairwise agreement (PPA/NPA) restricted to co-tested patients, minimizing denominator bias and avoiding overstating specificity in a case-only design.

Conventional methods remain indispensable despite recognized shortcomings. India ink staining, though low-cost and rapid, shows highly variable sensitivity (range, 42%–86%) and is operator-dependent (Stack et al., 2023). Alcian blue staining showed moderate yield in our cohort but, like India ink, requires sufficient organism load and technical expertise (Beardsley et al., 2019). Culture remains specific yet often turns negative after prior antifungal exposure or when burden is low (Howard-Jones et al., 2022). Capsular antigen testing is generally highly sensitive, but rare false-negatives occur with post-zone effects or capsule-deficient strains (Setianingrum et al., 2019). Within this context, mNGS added value by identifying Cryptococcus when culture was negative or pretreatment had occurred and by correcting a phenotypic misidentification.

As a hypothesis-free, pan-pathogen assay, CSF mNGS can concurrently detect viral, bacterial, fungal and parasitic pathogens, enabling recognition of mixed infections that may alter management (e.g., targeted antivirals or antibacterial coverage in addition to antifungals) (Gu et al., 2021; Diao et al., 2022). In our cohort, viruses (EBV/CMV/HIV-1) were identified in five patients by mNGS, findings that routine workflows did not systematically capture, underscoring mNGS as a single-assay complement to conventional testing in complex hosts. Published clinical series likewise show that CSF mNGS can broaden the diagnostic yield across pathogen classes and inform treatment decisions (Benoit et al., 2024).

Beyond species identification, strain/lineage resolution within the C. neoformans/C. gattii complex can inform epidemiology, prognosis and outbreak investigation. For example, the Vancouver Island outbreak was driven by C. gattii VGII lineages (notably VGIIa/”major”), with distinct ecological and clinical patterns across the Pacific Northwest (Kidd et al., 2004). While standard short-read clinical mNGS typically reports species level, integrating lineage-informative markers or targeted sequencing could enable genotype-aware surveillance (e.g., C. gattii vs C. neoformans lineages) and, where feasible, tracking of resistance-associated variants to support antifungal stewardship.

Practical adoption depends on cost, access, and turnaround time (TAT). CrAg LFA is inexpensive and point-of-care (≈ 5–15 min), supporting both screening (advanced HIV) and rapid rule-in; CSF culture provides specificity and susceptibility but may take days to weeks; current clinical CSF mNGS at reference centers reports a median TAT ≈ 9 days, though faster (≈1–4 days) has been achieved in dedicated single-center pipelines. A context-sensitive reflex algorithm could be: (i) CrAg-first in suspected CM; (ii) mNGS for discordant results (e.g., high clinical suspicion with negative/indeterminate CrAg or smear), atypical/complex hosts, suspected mixed infections, or when species/lineage resolution may influence management (e.g., unusual gattii-complex disease). Such algorithms align with guideline pathways and may improve yield without undue cost or delay (WHO Guidelines Approved by the Guidelines Review Committee, 2022).

This study has several limitations. First, the sample size was relatively small (n=23) in a university-affiliated tertiary care center setting, which potentially has affected the generalizability of the findings. Second, despite the prospective collection of data, some patients lacked complete conventional diagnostic data, limiting head-to-head method comparisons. Third, mNGS testing was not uniformly timed, with a wide range (10 to 415 days from symptom onset), potentially affecting sensitivity. Fourth, mNGS is known to be affected by prior antifungal treatment, which likely contributed to false negatives in several treated cases. Fifth, spinal or localized lesions may lead to CSF-negative results, as seen in Case 20, suggesting that sampling strategy is crucial for accurate detection. Finally, lack of quantitative fungal load assessment and follow-up data limited our ability to evaluate mNGS as a monitoring tool. Standardizing thresholding against batch-specific background, reporting of controls (NTCs), and integration of lineage markers may further improve interpretability. Prospective studies should evaluate patient-centered outcomes (time to directed therapy, complications, and mortality) and cost-effectiveness of reflex strategies in diverse settings.

In conclusion, mNGS enhances diagnostic evaluation of suspected CM by increasing detection of Cryptococcus and unveiling mixed infections. Its greatest value may lie in complex, atypical, or pretreated cases. Future large-scale multi-center studies are warranted to define standardized interpretation criteria, optimal timing, and cost−effective integration into clinical pathways.

## Data Availability

The raw data supporting the conclusions of this article will be made available by the authors, without undue reservation.
